# 2544. Population pharmacokinetic meta-analysis of BV100 in healthy subjects and renally impaired subjects

**DOI:** 10.1093/ofid/ofad500.2161

**Published:** 2023-11-27

**Authors:** Jon Moss, Michael Watts, Gitzinger Marc, Christian Kemmer, Lisa Husband, Glenn Dale

**Affiliations:** BAST, Kington, England, United Kingdom; BAST, Kington, England, United Kingdom; BioVersys AG, Basel, Basel-Stadt, Switzerland; BioVersys AG, Basel, Basel-Stadt, Switzerland; BioVersys SAS, Lille, Haute-Normandie, France; BioVersys AG, Basel, Basel-Stadt, Switzerland

## Abstract

**Background:**

We recently identified rifabutin as having potent antibacterial activity towards *Acinetobacter baumannii* under nutrient limiting conditions.

BV100 is the abbreviation for a novel intravenous formulation of rifabutin under development for the treatment of serious infections due to *A. baumannii*. The overall objective of this analysis was to use all available data to build a population pharmacokinetic model capable of describing the kinetics of BV100 after single and multiple IV infusions in healthy subjects and renally impaired subjects and to provide, through simulation of popPK models, estimates of efficacious doses of BV100.

**Methods:**

The analysis was conducted via nonlinear mixed-effects modelling using NONMEM (ICON Development Solutions, version 7.4). Data extraction, exploratory analysis, and all graphical displays and statistical tests were performed in R version 4.1.2. Simulations for visual predictive checks (VPCs) were executed using NONMEM. Exposure simulations were accomplished in R using the “RxODE” library. Simulations of the final popPK model were conducted to directly compare model-predicted Cmax and AUC(0-24) between healthy subjects and subjects with renal impairment.

Simulations of the final popPK model were done to identify the dose at which subjects of varying degrees of renal impairment were expected to achieve target exposures above specified threshold values for 1-log reduction in bacteria at MIC values of 0.5, 1 and 2 mg/L.

**Results:**

A total of 1740 PK samples from 95 subjects were used in the final popPK model inference. A three compartment popPK model with inter-individual variability (IIV) on clearance, volumes of the central compartment (V1), peripheral compartment (V2) and the inter-compartmental clearance (Q3) produced predictions in agreement with measured BV100 plasma concentrations. Model simulations predict that at steady state, q12h dosing of 150, 250 and 525 mg is sufficient for 90% of subjects with any level of renal impairment to achieve rifabutin exposures expected to achieve 1-log reduction in bacterial load at MIC values of 0.5, 1 and 2 mg/L, respectively.

**Conclusion:**

The PK of intravenous BV100 is adequately described by a three-compartment linear popPK model.

**Disclosures:**

**Gitzinger Marc, PhD**, BioVersys AG: Ownership Interest **Christian Kemmer, PhD**, BioVersys AG: Ownership Interest **Lisa Husband, MBBS BSc MRCPCH**, BioVersys AG: Ownership Interest **Glenn Dale, PhD**, BioVersys AG: Ownership Interest

